# Recommended Initial Antimicrobial Therapy for Emphysematous Pyelonephritis: 51 Cases and 14-Year-Experience of a Tertiary Referral Center

**DOI:** 10.1097/MD.0000000000003573

**Published:** 2016-05-27

**Authors:** Yu-Chuan Lu, Jian-Hua Hong, Bing-Juin Chiang, Yuan-Hung Pong, Po-Ren Hsueh, Chao-Yuan Huang, Yeong-Shiau Pu

**Affiliations:** From the Department of Urology (Y-CL), National Taiwan University Hospital, Yunlin Branch ; Department of Urology (J-HH, B-JC, Y-HP, C-YH, Y-SP), National Taiwan University Hospital; Departments of Laboratory Medicine and Internal Medicine (P-RH), National Taiwan University Hospital, National Taiwan University College of Medicine, Taipei, Taiwan.

## Abstract

The aim of this study was to investigate the profiles of pathogens and patterns of antibiotic resistance of emphysematous pyelonephritis (EPN), offering recommendations for initial antibiotic treatment.

Between January, 2001, and November, 2014, demographic data, presenting clinical features, management strategies, and treatment outcomes of 51 patients with EPN were retrospectively reviewed, analyzing microbiological characteristics of causative pathogens and patterns of antibiotic resistance.

Overall survival rate was 90.2% (46/51). Pathogens isolated most frequently were *Escherichia coli* (49.0%), *Klebsiella pneumoniae* (19.6%), and *Proteus mirabilis* (17.7%). Approximately 24% of *E coli* isolates and 22% *K pneumoniae* isolates were resistant to fluoroquinolones. Improper empiric antibiotic use (*P* = 0.02) and third-generation cephalosporin-resistant pathogens (G3CRP) (*P* = 0.01) were significantly more common in cases of patient fatality. Prior hospitalization and antibiotic use within past year (*P* = 0.03), need for emergency hemodialysis (*P* = 0.03), and development of disseminated intravascular coagulation (DIC) (*P* = 0.03) were factors correlating significantly with microbial resistance to third-generation cephalosporins. The area under the receiver operating characteristic curve was 0.91. The cut-off point determined by the maximum Youden index for 2 of these 3 factors yielded a sensitivity of 0.8 and specificity of 0.93.

Third-generation cephalosporins are recommended as initial treatment of EPN. In patients with histories of prior hospitalization and antibiotic use and in those needing emergency hemodialysis or developing DIC, carbapenem is the empiric antibiotic of choice. Patients presenting with 2 or more factors carry the highest risk of G3CRP involvement. Fluoroquinolone and gentamicin should be avoided.

## INTRODUCTION

Emphysematous pyelonephritis (EPN) is a rare but life-threatening acute necrotizing infection of the kidney that generates gas within the renal parenchyma/collecting system or perinephric tissues.^1,2^ Schultz and Klorfein (1962) reported the first case series of EPN, although the term “emphysematous pyelonephritis” was first applied by Schultz and Klorfein (1962).^3^ This illness is generally perpetrated by enteric gram-negative facultative anaerobes (such as *E coli*, *Klebsiella spp.,* and *Proteus spp.*), which ferment glucose and lactate to carbon dioxide, producing necrosis.^4^ It occurs most frequently in female diabetic patients (70–90%) and carries a mortality rate of up to 80%, if patients are only treated medically.^1,5^

Timely initiation of suitable antibiotics and percutaneous catheter drainage (PCD) are of utmost importance as treatment.^5,6^ To maximize nephron sparing, PCD has been widely adopted and in conjunction with medical treatment has succeeded in lowering the mortality rate to 13.5%.^4^ Any empiric antimicrobial regimen should primarily target gram-negative bacteria. Existing protocols for prophylaxis in this setting usually incorporate β-lactamase inhibitors, cephalosporins, aminoglycosides, and quinolones, according to smaller studies.^6,7^ However, the validity of such policies may be fleeting, given the global rise in antimicrobial resistance.

Previous investigations of patients with EPN have focused on prognostic factors and determining a gold standard of decisive management for the majority of patients. However, none to date has fully delineated optimal antimicrobial therapy or provided a novel management algorithm for EPN.

The aim of this study was to investigate microbiological profiles of culpable uropathogens and determine prevailing patterns of antibiotic resistance, offering recommendations for initial antimicrobial therapy of patients with EPN.

## METHODS

### Patients and Setting

Between January, 2001, and November, 2014, 51 consecutive patients with EPN received treatment at the National Taiwan University Hospital, a tertiary referral center in Taiwan. Patient demographics and other variables, including age, gender, underlying medical conditions, laboratory findings, presenting clinical features, interval between symptoms and start of treatment, imaging study results, and treatment outcomes, were acquired from medical charts. Clinical features consisted of signs and symptoms at presentation, in addition to hemodynamic indices and mental status. Laboratory diagnostics (generally obtained at presentation) included white blood cell count, platelet count, C-reactive protein, serum albumin, serum sodium, hemoglobin A1C (HbA1c), and serum creatinine, as well as urinalysis and culture (blood, wound, and urine) results. This study was approved by the Institutional Review Board and Ethics Committee of National Taiwan University Hospital. According to the provisions of the Institutional Review Board of National Taiwan University Hospital, informed consents were not required for this pure retrospective chart review study. Patient records/information was anonymized and de-identified prior to analysis.

Initial EPN management strategies included antibiotics only, PCD with antibiotics, indwelling double-J catheter insertion with antibiotics, and emergency nephrectomy. Empiric antibiotic treatment was deemed appropriate when found to match in vitro susceptibilities of corresponding pathogens. Salvage nephrectomy or open drainage was done if PCD or conservative treatment failed.

## DEFINITIONS

Each patient was assigned to 1 of the 5 categories stipulated by Huang and Tseng,^8^ reflecting extent of gas seen on computed tomography (CT), as follows: class 1, collecting system only; class 2, renal parenchyma only, without involvement of extrarenal space; class 3A, extension to perinephric space (gas or abscess); class 3B, pararenal space extension (gas or abscess); and class 4, bilateral or whole-kidney EPN.

Thrombocytopenia was equated with a platelet count < 120,000/mL, severe hypoalbuminemiawith a serum albumin < 3.0 g/dL, and hyponatremia with a serum sodium < 135 mEq/L. Shock was defined as a systolic blood pressure < 90 mm Hg, with evidence of end-organ damage (i.e., lungs, liver, or kidneys). Patients with absolute increases in serum creatinine ≥0.3 mg/dL after admission (vs baseline level) were diagnosed with acute kidney injury. The diagnosis of disseminated intravascular coagulation (DIC) was based on the ISTH Diagnostic Scoring System for DIC. Failure of initial treatment was defined as intra-hospital mortality, appearance of unstable hemodynamics, or a prolonged fever after initial treatment within 7 days.

Using the disk diffusion method, in concert with guidelines of the Clinical and Laboratory Standards Institute (2006), uropathogen susceptibilities to the following agents were determined: (1) ampicillin; (2) gentamicin; (3) cefazolin; (4) second-generation cephalosporins (cefuroxime, cefoxitin, or cefmetazole); (5) third-generation cephalosporins (cefotaxime, ceftriaxone, ceftazidime, or cefixime); (6) fourth-generation cephalosporins (cefepime or cefpirome); and (7) fluoroquinolones (ciprofloxacin or levofloxacin). Intermediate susceptibility was considered as resistance.

### Statistical Analysis

Categorical variables were compared via chi-square or Fisher's exact test. Continuous variables were expressed as median values, and the Wilcoxon rank sum test was engaged for comparisons. Statistical significance was set at *P* ≤0.05. Variables with a *P* <  0.05 underwent receiver operating characteristic (ROC) curve analysis to determine their sensitivity and specificity for predicting third-generation cephalosporin-resistant pathogens (G3CRP) involvement.

## RESULTS

Table 1 summarizes clinical and epidemiologic characteristics of patients with EPN and all related microbiologic profiles. Based on CT imaging (data of 2 patients missing), EPN class distributions were as follows: class 1, n = 10; class 2, n = 14; class 3A, n = 11; class 3B, n = 10; and class 4, n = 4 (including 2 EPN of a solitary kidney and 2 bilateral involvement).

**TABLE 1:**
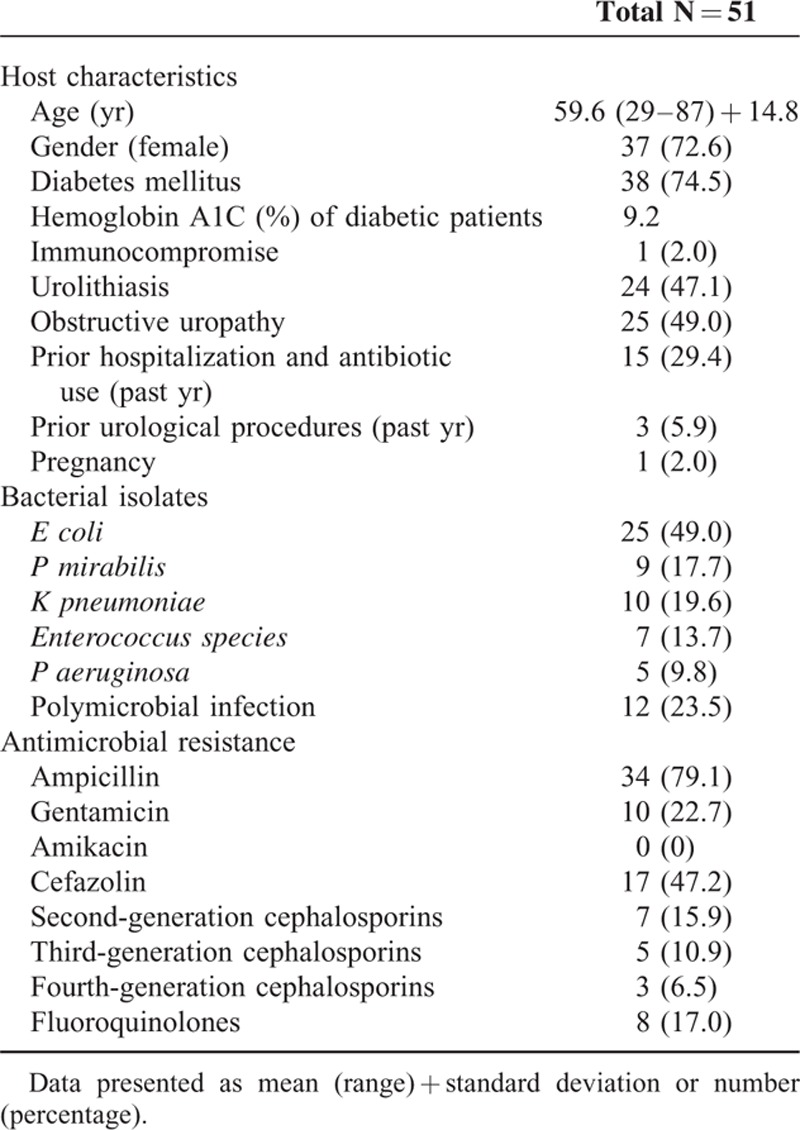
Patient Demographics and Microbiological Characteristics in Emphysematous Pyelonephritis

Overall survival rate was 90.2% (46/51). Altered mental status was evident in 17 patients, and 11 patients presented with shock. Another 28 patients qualified as acute kidney injury. Three of eight patients requiring emergency hemodialysis died. Mean leukocyte count during admission was 15683/μL. Thrombocytopenia and hypoalbuminemia were noted in 15 (29.4%) and 27 (52.9%) patients, respectively. Ten patients received antibiotics alone, and treatment was successful in 80.0% of these patients (2 patients died). Most of the cases were limited EPN (class 1 and 2), comparatively healthy and suffered from EPN a decade ago. Six patients had class 1 EPN, 3 had class 2, and 1 had class 3A with a small air pocket. One patient with class 1 EPN caused by ureteral stone underwent ureteroscopy with double-J catheter stenting and survived. Thirty-nine patients were treated with PCD. One patient underwent emergent nephrectomy and survived. However, 5 patients received salvage nephrectomy and 1 underwent open drainage as a result of failure of initial treatment.

**FIGURE 1:**
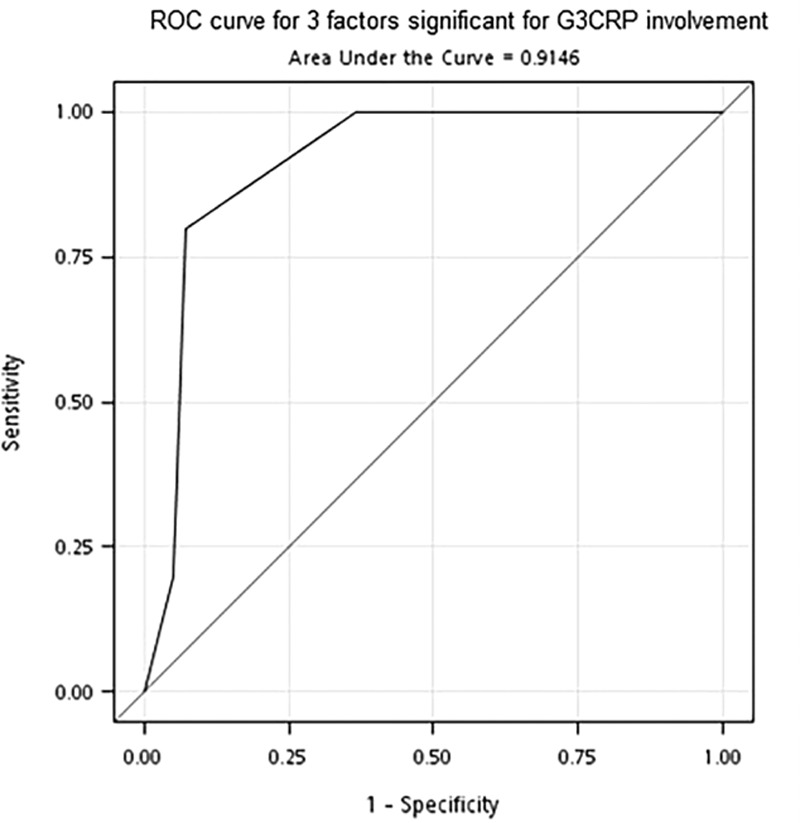
ROC curve for 3 factors significant for G3CRP involvement. G3CRP = third-generation cephalosporin-resistant pathogens, ROC = receiver operating characteristic.

Need for emergency hemodialysis (*P* = 0.02), shock at presentation (*P* = 0.01), altered mental status (*P* = 0.04), third-generation cephalosporin-resistant pathogen (G3CRP) (*P* = 0.01), improper empiric antibiotic usage (*P* = 0.02), and polymicrobial infection (*P* = 0.001) were significantly more likely in cases where fatalities occurred (Table 2). Outcomes (survival vs death) did not differ significantly in terms of age, history of diabetes, organisms implicated, thrombocytopenic status, acute kidney injury rate, serum creatinine level, and leukocyte count or presence of microscopic hematuria, pyuria, urinary tract obstruction, urolithiasis, the management modality (*P* = 0.66), and interval between symptoms and start of treatment. However, prior hospitalization and antibiotic use within past year (*P* = 0.03), need for emergency hemodialysis (*P* = 0.03), and development of DIC (*P* = 0.03) correlated significantly with G3CRP (Table 3). These 3 variables were evaluated by ROC analysis and the AUC; sensitivity and specificity were estimated for each variable. The AUC of the ROC curve for the 3 factors was 0.91 (Figure 1). The optimal cut-off value for 2 variables was based on the highest Youden index (0.73) and resulted in a sensitivity of 0.8 and specificity of 0.93 for predicting a G3CRP involvement.

**TABLE 2:**
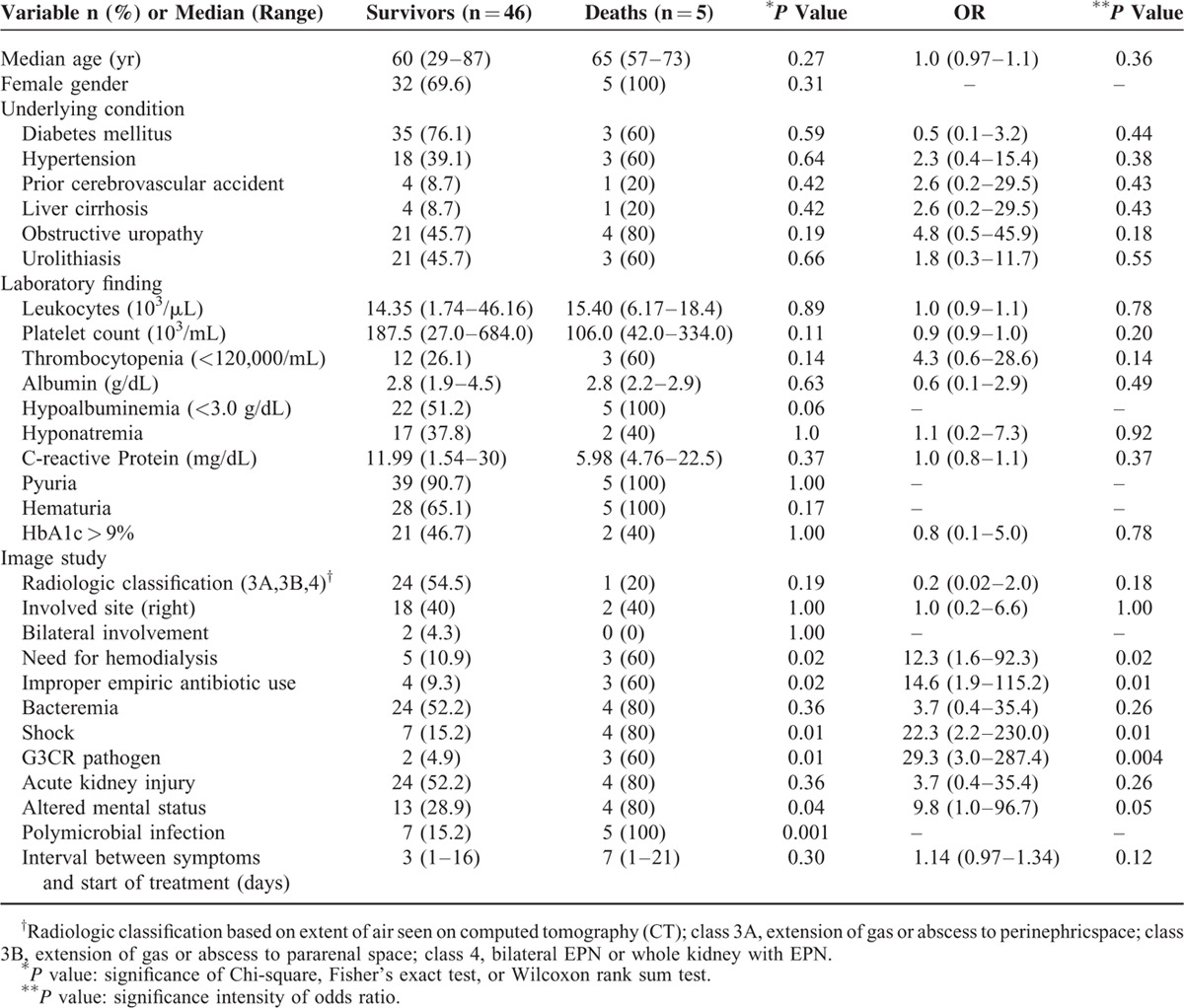
Patient Parameters in Emphysematous Pyelonephritis by Outcomes (Survival vs Death)

**TABLE 3:**
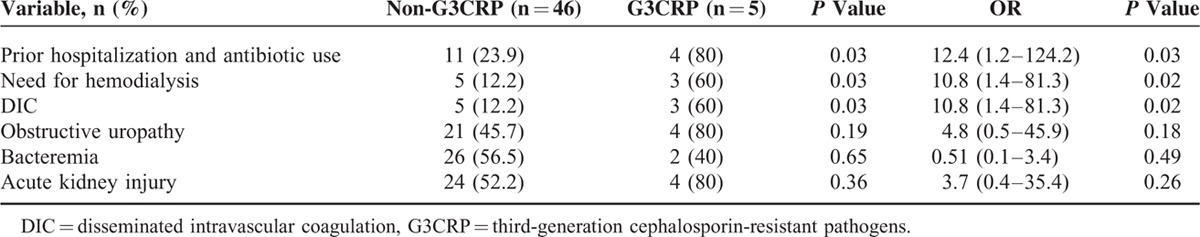
Factors Correlating With Third-Generation Cephalosporin-Resistant Pathogens (G3CRP)

*E coli* was the organism most frequently isolated from urine, blood, or wound cultures, identified in 25 patients (49.0%). Other isolates included *K pneumoniae* (19.6%), *P mirabilis* (17.7%), *Enterococcus species* (13.7%), and *P aeruginosa* (9.8%). Polymicrobial infections were found in 12 patients (23.5%). Bacteremia occurred in 28 patients (54.9%), attributable to *E coli* in 15 patients.

Third-generation cephalosporins (20/51, 39.2%) were the initial drugs of choice in most cases. Treatment was adjusted as needed, once results of cultures became available. The following overall antimicrobial resistance rates were determined: ampicillin, 79.1%; gentamicin, 22.7%; cefazolin, 47.2%; second-generation cephalosporins, 15.9%; third-generation cephalosporins, 10.9%; fourth-generation cephalosporins, 6.5%; and fluoroquinolones, 17.0%. Five patients harbored G3CRP isolates (*E coli*, n = 2; *K pneumoniae*, n = 1; *Stenotrophomonasmaltophilia*, n = 1; and *P aeruginosa*, n = 1), 3 of whom died. In vitro susceptibilities of pathogens cultured are detailed in Table 4.

**TABLE 4:**
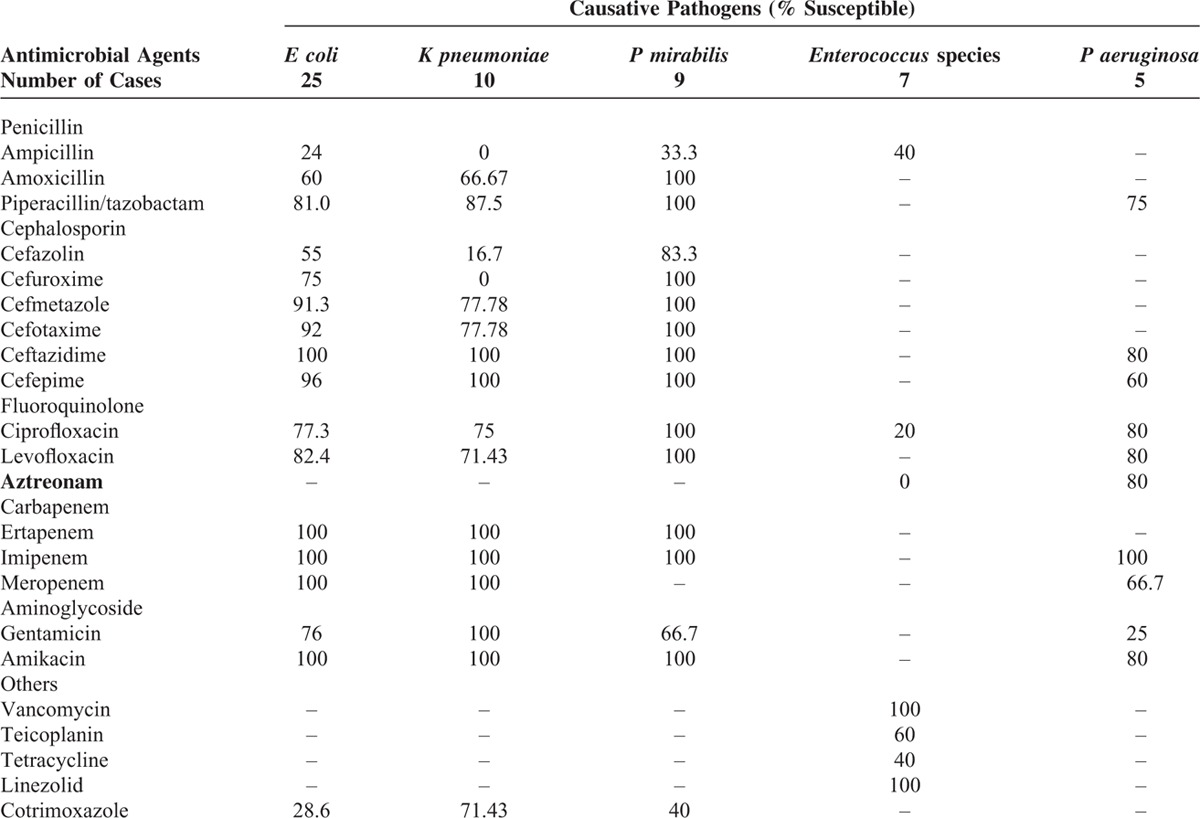
Antimicrobial Susceptibility Rates of Pathogens by the Disk-Diffusion Method

## DISCUSSION

The first step in managing a patient with EPN is fluid and electrolyte resuscitation, acid base balance, diabetic control, and an antibiotic regimen.^7^ A spectrum of management strategies for EPN has evolved over the years, ranging from invasive surgery to more conservative measures, including percutaneous catheter drainage (PCD) or placement of a double-J catheter (DBJ). Timely administration of appropriate antibiotics and early PCD are of paramount importance.^4^ To the best of our knowledge, this is the first study to address microbiological profiles and patterns of antibiotic resistance in EPN. Our recommendations for initial antibiotic use and a novel algorithm for managing patients with EPN are offered as well.

Empiric antibiotic treatment has been shown to reduce mortality in cases where gram-negative infections prevail.^9^ Such therapy should be broad spectrum (based on local antibiograms) and individualized (attuned to patient characteristics and antimicrobial resistance), taking into account severity of infection and patient vulnerability.^10,11^ In light of our results, initial therapy for EPN should primarily target *E coli*, *K pneumoniae*, and *P mirabilis*. Other organisms to consider are *P aeruginosa* and *Enterococcus* species.

Preferred single-agent options for treating EPN, effective against the highest percentage of bacterial isolates, are third- or fourth-generation cephalosporins (e.g., ceftazidime) and carbapenems. Alternate empiric regimens include a combination of amikacin and third-generation cephalosporin, given the very low overall resistance rates among *E coli*, *K pneumoniae,* and *P mirabilis*. Aminoglycosides must be used with care in patients with impaired renal function. The addition of gentamicin may be inappropriate and ineffective in this setting.

In patients who have been hospitalized and treated with antibiotics within the past year, who need emergency hemodialysis, or who have developed DIC, it is likely that a G3CRP is involved. The AUC of the ROC curve for the 3 significant factors was 0.91 and had statistically significant power to predict G3CRP involvement. Among the 7 patients with 2 or more factors, G3CRP was present in 4 cases (57%). Hence, empiric carbapenem treatment should be considered. As cited in other studies,^12,13^ prior antibiotic treatment is a known risk factor for third-generation cephalosporin-resistant *Enterobacteriaceae*. Extended spectrum beta-lactamase (ESBL) producing or Amp-C harboring isolates should be considered. First-choice therapies are also apt to be inadequate for patients with bacteremia due to ESBL producers, with devastating impact on clinical outcomes.^14^

Several international guidelines currently recommend fluoroquinolones as empiric treatment of choice for UTI.^15,16^ Nevertheless, 1 particular study (1999–2000) of community-associated UTI in adults found that nonbacteremic UTI due to *E coli* carries a 17% resistance rate to ciprofloxacin ^17^, and another investigation of levofloxacin susceptibility in clinical urinary *E coli* isolates, collected at 12 major teaching hospitals in differing parts of Taiwan, yielded rates in the range of 70% to 80%.^18^ These rates are aligned with our results. However, resistance of uropathogens to fluoroquinolones is increasing, which is a major clinical concern. Risk factors for fluoroquinolone-resistant *E coli* infection include recent hospitalization, prior fluoroquinolone use, and urinary catheter placement.^19–22^ Consequently, fluoroquinolones should be avoided as empiric treatment of EPN, given any of the risk factors above. Indiscriminate use of fluoroquinolones for complicated or catheter-related UTI may even undermine the susceptibility of respiratory pathogens to these agents.

In 2002, Huang and Tseng devised a 4-tier classification of EPN, based on the extent/distribution of gas seen by CT.^6^ Ultimately, they observed that mortality and PCD failure rates were associated with EPN of higher class (i.e., class 3 and 4 disease), giving patients with class 1 EPN the best prognosis. Still, several large studies have found no association between radiologicalEPN class and survival.^23–25^ Kapoor et al instead have claimed that degree of renal damage, rather than extent of gas on CT, is predictive of the need for nephrectomy.^23^ Consistent with earlier efforts, we found no statistically significant difference in mortality rates of class 3 and class 4 CT images. Thus, classification of EPN seems to have no real predictive value, perhaps owing to current treatment practices.

Various prognostic factors for mortality have been recognized. Huang and Tseng proposed that thrombocytopenia, disturbance of consciousness, severe proteinuria, shock, and acute renal failure were associated with a poor outcome.^8^ Khaira et al reported that shock was an independent predictive factor for mortality.^25^ Correspondingly, a study composed of 39 patients with EPN demonstrated that altered mental status, thrombocytopenia, renal failure, and severe hyponatremia at presentation were associated with higher mortality rates.^23^ However, none of the investigations studied a large population with prospective design to identify the most valuable prognostic factors. In the present study of 51 patients with EPN, need for emergency hemodialysis, shock at presentation, altered mental status, third-generation cephalosporin-resistant pathogen, improper empiric antibiotic usage, and polymicrobial infection were significantly associated with mortality. Although the factors, such as obstructive uropathy, female gender, thrombocytopenia and hypoalbuminemia, bacteremia and acute kidney injury showed no statistical association with mortality, they were seen in most of the nonsurvivors. Clinical importance of these factors should be taken into consideration.

**FIGURE 2:**
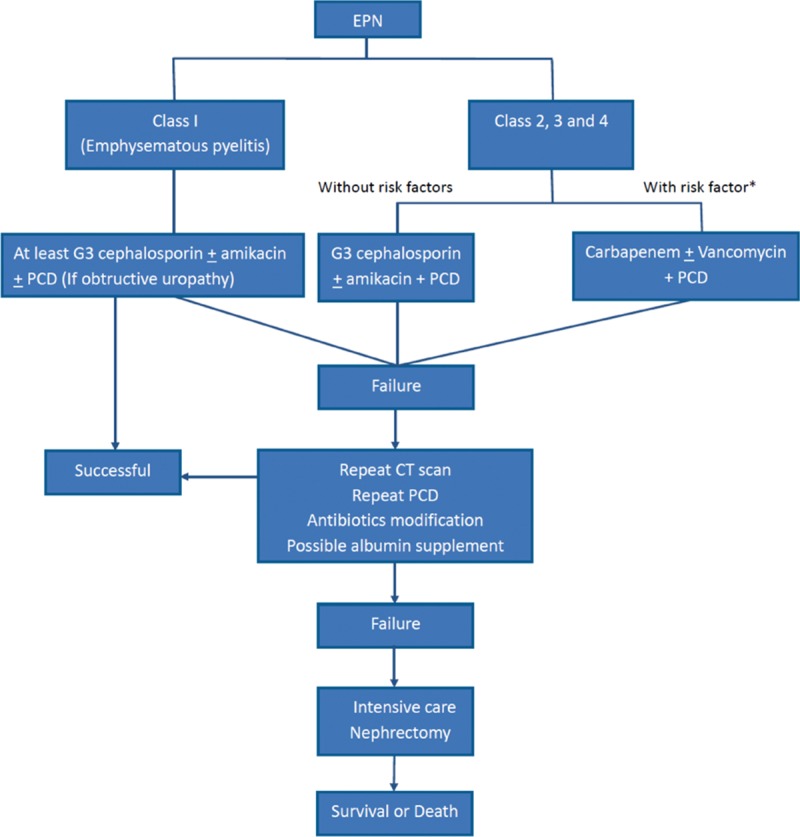
Algorithm for management of EPN. EPN = emphysematous pyelonephritis.

A novel algorithm for managing the typical patient with EPN is diagramed in Figure 2. During the past decade, there has been a steady shift in strategy toward nephron-sparing through PCD, with or without elective nephrectomy at a later date.^4^ Although it is tempting to suggest that PCD be attempted in all patients upfront,^6^ a small subset of patients will experience persistent fever or unstable hemodynamics in the aftermath of PCD. In this event, we strongly recommend that CT imaging and PCD be repeated and antibiotic treatment be modified. Albumin supplementation may also be beneficial. A prior study of ours showed that severe hypoalbuminemia (<3.0 g/dL) and increased risk of PCD failure were independently linked. However, the merit of correcting hypoalbuminemia at this juncture has not been clearly established. In this cohort, only 1 patient with class 1 EPN underwent ureteroscopy with double-J catheter stenting after a period of antibiotics treatment. Most of the EPN patients were critically ill and high risk of anesthesia at the early presentation that URS and DBJ stenting may not be appropriate as initial therapy. Due to limited evidence supporting and high risk of general anesthesia, we recommend PCN first.

This study has limitations inherent in all retrospective and single-center studies. The small patient sampling may explain the lack of statistical significance seen in various parameters. In addition, we are unable to stipulate an ideal duration of antimicrobial treatment and definitively specify a follow-up period. We also have difficulty offering an analysis of bacterial clonotypes, to address if these are unique strains in this geographic area. Baseline data obtained at patient presentation was largely used to identify risk factors for mortality. Such factors may have changed in the course of treatment, thus influencing outcomes. A larger prospective study is warranted to support our findings.

Use of an appropriate empiric antibiotic, typically a third-generation cephalosporin, and PCD are essentials in treating patients with EPN. In patients with histories of prior hospitalization and antibiotic use and in those requiring emergency hemodialysis or experiencing DIC, carbapenemis the empiric antibiotic of choice. Patients presenting with 2 or more factors carry the highest risk of G3CRP involvement. Fluoroquinolones and gentamicin should be avoided.
